# RNA-Sequencing in Resistant (QL3) and Susceptible (Theis) Sorghum Cultivars Inoculated With Johnsongrass Isolates of *Colletotrichum sublineola*

**DOI:** 10.3389/fgene.2021.722519

**Published:** 2021-08-11

**Authors:** Purushothaman Natarajan, Ezekiel Ahn, Umesh K. Reddy, Ramasamy Perumal, Louis K. Prom, Clint Magill

**Affiliations:** ^1^Department of Biology, Gus R. Douglass Institute, West Virginia State University, West Virginia, WV, United States; ^2^Department of Plant Pathology and Microbiology, Texas A & M University, College Station, TX, United States; ^3^Agricultural Research Center, Kansas State University, Hays, KS, United States; ^4^Crop Germplasm Research Unit, USDA-ARS Southern Plains Agricultural Research Center, College Station, TX, United States

**Keywords:** RNASeq, sorghum, anthracnose, Johnsongrass, differentially expressed genes

## Abstract

Gene expression was analyzed at 0- and 24-h post-inoculation of two inbred sorghum cultivars known to differ in response to inoculation with *Colletotrichum sublineola*, the fungal pathogen that causes anthracnose. QL3 is reported to have quantitative resistance, while Theis is susceptible to most pathotypes of the pathogen; RNASeq identified over 3,000 specific genes in both cultivars as showing significant changes in expression following inoculation; in all but one gene, the changes in QL3 and Thies were in the same direction. Many other genes showed significant changes in only one of the two cultivars. Overall, more genes were downregulated than upregulated. Differences in changes in expression levels of a few genes suggested potential roles for the difference in disease response between QL3 and Theis, but did not identify known resistance genes. Gene ontology (GO) and pathway enrichment analysis identified upregulation of 23 transcription factor encoding genes as well as genes involved in the production of secondary metabolites, which are part of a typical host defense reaction.

## Introduction

Sorghum [*Sorghum bicolor* (L.) Moench] is a crop grown for both grain and stover. Water use efficiency makes sorghum especially important in drier climates. On the other hand, the close relative of sorghum, Johnsongrass [*Sorghum halepense* (L.) Pers.], is considered one of the most noxious weeds and has adapted to colder climates is now found on a global scale (Fletcher et al., 2020). Estimated to have evolved separately for at least years (Paterson et al., 2020), Johnsongrass, a tetraploid that originated from a cross between the diploid species *S. bicolor* and *S. propinquum*, can cross hybridize with *S. bicolor*, especially if the sorghum is male sterile (Ohadi et al., 2017). The similarity of their genomes raises concern that Johnsongrass may serve as a reservoir for diseases of sorghum. This is tempered somewhat by the possibility that it may also provide sources for disease resistance.

Anthracnose, caused by *Colletotrichum sublineola* P. Henn, in Kabat and Bubák, is one of the most damaging diseases of sorghum in most the growing areas in the world (Prom et al., 2015). The fungus can infect the leaves, talks, and panicles, although, foliar infection is most common. Symptoms may appear as small circular to elliptical spots or elongated lesions, and as the fungus sporulates, fruiting bodies (acervuli) appear as black spots in the center of the lesions (Thakur and Mathur, 2000). Estimating grain yield losses due to foliar anthracnose infection can often be difficult, but losses as high as 50% have been reported in susceptible cultivars (Prom et al., 2015). The pathogen can be isolated from both Johnsongrass and sorghum. At least so far as sorghum is concerned, there is tremendous variability in *C. sublineola*, including pathogenicity. This may well be associated with breeding efforts to select resistance, which often have resulted in selecting for single genes that are soon overcome by adaptations in the pathogen. As an example, Thies, a cultivar used in this study, was released in 1974 as a source of anthracnose resistance (Broadhead et al., 1978), but in 2012 screens, it was susceptible to eight of 10 pathotypes identified by tests using 12 differentials (Prom et al., 2012). Resistance in cultivar QL3 to all isolates of the pathogen tested from the US and Puerto Rica has been found to be a quantitative trait (Vermerris et al., 2018). As would be expected, little information is available concerning the variability of pathogenic races among Johnsongrass isolates. Xavier et al. (2018) found only four of 10 isolates from *S. halepense* caused disease on any of 13 *S. bicolor* accessions, while Ahn et al. (2020) found that one of three sorghum isolates from sorghum tested *via* a detached leaf assay led to pathogen reproduction in all 26 Johnsongrass cultivars tested at the eight leaf stage. Ahn et al. (2020) also found that spraying Johnsongrass after the heading stage, but not at the five or eight leaf stage with that same isolate, did give disease, as demonstrated by the formation of acervuli. Personal observations of the consequences of spraying sorghum cultivars with 10^6^ spores/ml of Johnsongrass isolates revealed high levels of hypersensitive response (HR), but relatively few cases of actual disease in terms of fungal reproduction were noted.

Inoculation of sorghum with *C. sublineola* has been shown to rapidly induce the production of pigmented phytoalexins (Lo et al., 1999), and confirmation of induction of mRNAs for phenylalanine ammonia lyase (PAL) and chalcone synthase, enzymes required for making these compounds was demonstrated by northern blots (Cui et al., 1996). Recently, the response to inoculation with *C. sublineola* has been measured in two large and diverse sorghum collections of accessions referred to as the minicore (Upadhyaya et al., 2009) and the Sorghum Association Panel (SAP; Casa et al., 2008). Since whole-genome sequencing data for these accessions, among others, has been made publicly available by Dr. Geoff Morris^1^ (Morris et al., 2013) it has been possible to use Genome-Wide Association Studies (GWAS) to tag genes by identifying Single Nucleotide Polymorphisms (SNP) alleles associated with a variety of traits. In the case of anthracnose, SNPs associated with resistance and susceptible cultivars have been identified for isolates from Puerto Rico (Cuevas et al., 2018, 2019) and Texas (Ahn et al., 2019; Prom et al., 2019). In almost all cases, the gene nearest a high scoring SNP has been shown to have a role in host defense in other hosts/pathogen interactions, thus identifying over 30 genes that are likely to also be important in sorghum.

In this study, we have used RNASeq to investigate differences in gene expression in early defense responses of two sorghum cultivars that differ significantly in anthracnose sensitivity to try to pinpoint genes and pathways that may contribute to that difference, and to compare results with another very recent publication that did similar experiments but with emphasis on microRNAs with a different pair of anthracnose resistant and susceptible cultivars (Fu et al., 2020). Here, a mix of two *C. sublineola* isolates from Johnsongrass that had previously been shown lead to different visible responses on QL3 and Theis were used to examine gene expression changes in the two cultivars 24 h post-inoculation.

## Materials and Methods

### Collection of Plant Materials and Inoculation

Sorghum cultivars QL3 (resistant) and Theis (susceptible to most pathotypes *of C. sublineola*; Prom et al., 2012) were selected for the study and grown in the greenhouse to the eight-leaf stage. The plants were then transferred to a controlled growth chamber in the Plant Pathology building for inoculation and sampling. Two aggressive isolates of *C. sublineola* JG16081 and JG16083 were obtained from Dr. Gary Odvody at the Texas Agrilife Research and Extension Center, Beaumont, Tx. A 1:1 mixture of spores of these two isolates collected following growth on ½ strength PDA and adjusted to 10^6^/ml of water with a drop of Tween20 as a wetting agent was used for inoculum. Spores were applied to pre-marked segments on leaves of three plants of each cultivar. Zero-time samples were collected and frozen immediately in liquid nitrogen and stored at −70°C before the RNA isolation. At 24 h, the inoculated leaf segments were collected in the same manner.

### Total RNA Isolation and RNA-Seq Library Preparation

Total RNA from the leaf tissues of three biological replicates was isolated using RNeasy Plant Mini Kit (Omega Bio-tek, United States). The RNA was treated with DNAseI (Qiagen, United States) to remove co-isolated genomic DNA and subsequently purified using RNeasy MinElute Cleanup Kit (Qiagen, United States). The quality and quantity of the total RNA were analyzed using Agilent 2100 Bioanalyzer and Qubit 4 Fluorometer (Invitrogen, United States) instruments, respectively. According to the manufacturer’s protocol, RNA sequencing libraries were prepared using NEBNext® Ultra™ II RNA Library Prep Kit (NEB, United States). The mRNAs were enriched from total RNA using magnetic beads with Oligo (dT), and subsequently, they were fragmented into shorter fragments using fragmentation buffer. The first-strand cDNA was synthesized from the fragmented mRNA using random hexamer primers and converted into double-strand cDNA. The resulting double-strand cDNAs were end-repaired, and Illumina sequencing adapters were added. The adapter-ligated libraries were amplified using sequencing primers for enrichment. The library’s quality and insert size were determined using a bioanalyzer (Invitrogen, United States), and the library was quantified using fluorometric analysis (Invitrogen, United States). The library was diluted to 4 nM concentration and sequenced using Illumina’s NextSeq 500 platform with paired-end sequencing chemistry. The resulting image files in the bcl format were converted to FASTQ with 2 × 75 bp reads using the bcl2fastq tool (Illumina, United States).

### RNA-Seq Data Analysis

The sequencing adapters and the low-quality reads (Phred score QV < 30) were filtered using the read trimming tool Trimmomatic v.0.39 (Bolger and Giorgi, 2014). The quality-filtered reads were mapped to the *S. bicolor* reference genome build “assembly Sorghum_bicolor_NCBIv3” (Cooper et al., 2019) using STAR RNA-Seq aligner v.2.7.9 (Dobin et al., 2013) to generate alignments. The read count table was created using BAM alignment, general feature format (GFF) of genome annotation, and HTSeq v.0.13.5 (Anders et al., 2015). The differentially expressed genes (DEGs) among different experimental pair-wise combinations were identified using the DESeq2 R package (Love et al., 2014). The DEGs were filtered based on the minimum log_2_Fold Change of 1 and false discovery rate (FDR) of 0.05. Gene Annotation and GO Enrichment analysis were performed using Omicsbox.^2^ Pathway mapping was performed using the KEGG database, MapMan v.3.6.0 (Thimm et al., 2004; Schwacke et al., 2019) and Pathview web tool (Luo et al., 2017). In these analyses, *q* values were used to minimize the number of potential false positives associated with the use of *p* values (Storey and Tibshirani, 2003). Plant disease resistance genes were identified using the plant disease resistance genes database (Osuna-Cruz et al., 2018).

## Results

### Physiological Responses Observed

In order to determine susceptibility to the mixture of two isolates, greenhouse inoculation was conducted in 2017 and 2019 with 19 sorghum lines at eight leaf stage as described in Prom et al. (2012). Among the tested lines, QL3 was evaluated to be one of the most resistant lines, while Theis was scored to be the most susceptible line. Even though, we describe sorghum lines as susceptible based on necrosis on leaves, acervuli formation was not seen during greenhouse inoculations. At 7 days post-inoculation (dpi), small necrotic lesions were shown on a few leaves in QL3, while Theis showed severe necrotic lesions. By 28 days, the differences were dramatic, as shown in Supplementary Figure 1.

### RNA Sequencing and Identification of DEGs

RNA sequencing was used to identify the transcriptome response to the anthracnose infection in resistant (QL3) and susceptible (Theis) cultivars of Sorghum. The transcriptome analysis was performed with the samples at 0 and 24 h after infection (hpi). A total of 321, 992, 578 reads were obtained, which included 281,724,736 quality-filtered reads. The filtered reads were aligned to the Sorghum reference genome with an average mapping percentage of 88.4%. A summary of the RNA-Sequencing and genome mapping is given in Table 1.

**Table 1:** Summary of RNA Sequencing from resistant (QL3) and susceptible (Theis) sorghum cultivars infected with Johnsongrass anthracnose.

Sample name	Time points	Total number of raw reads	Total number of quality filtered reads (Q30)	Number reads mapped to the Sorghum reference genome (percentage of reads)	Number of reads mapped to *Colletotrichum sublineola* genome (percentage of reads)
QL3_0h_1	0 h	24,822,576	22,148,930	20,109,776 (90.8%)	32,386 (0.15%)
QL3_0h_2	0 h	25,571,038	22,436,394	20,341,748 (90.7%)	41,648 (0.19%)
QL3_0h_3	0 h	24,777,366	21,381,502	19,611,264 (91.7%)	43,380 (0.20%)
QL3_24h_1	24 hpi	25,595,308	22,625,858	20,421,618 (90.3%)	33,788 (0.15%)
QL3_24h_2	24 hpi	29,825,024	26,387,346	23,465,666 (88.9%)	39,220 (0.15%)
QL3_24h_3	24 hpi	29,892,494	26,592,080	23,956,298 (90.1%)	36,014 (0.14%)
Theis_0h_1	0 h	25,534,524	22,660,668	20,642,302 (91.1%)	30,390 (0.13%)
Theis_0h_2	0 h	27,938,168	24,749,774	21,930,936 (88.6%)	38,618 (0.16%)
Theis_0h_3	0 h	27,755,892	24,134,358	21,644,166 (89.7%)	25,114 (0.10%)
Theis_24h_1	24 hpi	27,661,552	24,269,816	21,304,054 (87.8%)	28,094 (0.12%)
Theis_24h_2	24 hpi	24,185,876	19,145,006	14,475,832 (75.6%)	17,134 (0.09%)
Theis_24h_3	24 hpi	28,432,760	25,193,004	21,594,880 (85.7%)	28,896 (0.16%)

**Figure 1:**
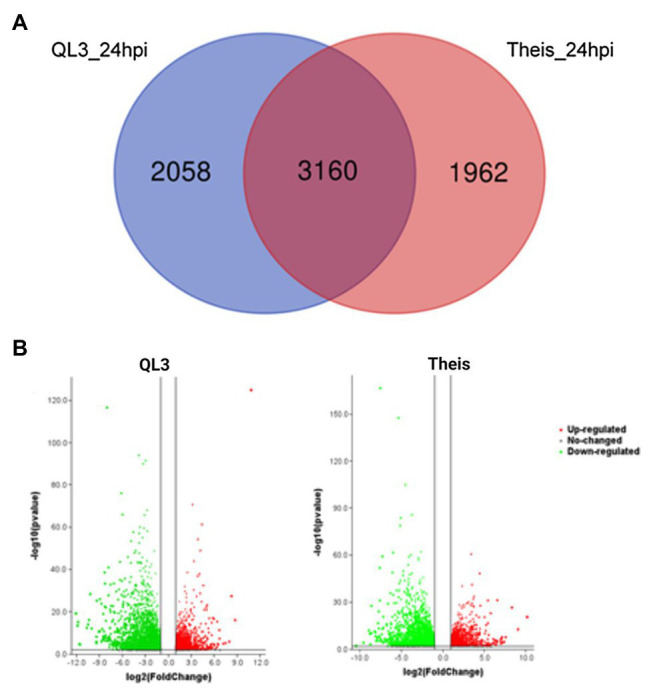
**(A)** Venn diagram showing the number of differentially expressed genes (DEGs) among different pair-wise experimental combinations infected with Johnsongrass anthracnose. **(B)** Volcano plot showing the DEGs from QL3 and Theis. Green represents downregulated genes and red represents upregulated genes.

The number of genes showing DEGs between the 0 and 24-h samples is shown in Table 2, where the cutoff used was a Log_2_fold change > 1. More DEGs for both the Resistant and Susceptible cultivars had decreased rather than increased expression. Comparison of the DEGs for the two hosts for samples taken 24 hpi showed that 3,160 were shared, while approximately 2,000 differed (Figure 1A). As shown in the volcano plot’ in Figure 1B, while most of the expression differences were close to the cutoff, a significant number had more than 5-fold changes, and even some of the more minor changes were highly significant, indicating consistency among replicates. Because exposure to pathogens in sorghum is known to involve the induction of defense-response genes such as those encoding enzymes, including those needed to make PAL-pathway derived phytoalexins and chitinase that can degrade fungal cell walls, emphasis was placed on identifying DEGs that are expressed to a higher degree in QL3 than Theis. A list of the top 25 of the highest scoring genes with increased expression 24 hpi in QL3 and the relative increase, if any in Theis, is shown in Table 3. A complete list of significantly up or downregulated DEGs in QL3 (resistant) and Theis are available in Supplementary Tables 1 and 2. DEGs common to both the hosts is available in Supplementary Table 3.

**Table 2:** Statistics of differentially expressed genes in resistant and susceptible cultivars.

Sample condition	Total number of DEGs	Upregulated DEGs	Downregulated DEGs
QL3 24 hpi	5,218	1,986	3,232
Theis 24 hpi	5,122	1,972	3,150

**Table 3:** Top 25 DEGs upregulated in the resistant genotype QL3 by anthracnose infection and relative up or downregulation in Theis.

Gene ID	Annotation	*Arabidopsis* homolog	Log_2_fold change QL3/Thies
LOC110430279	Lichenase-2 precursor	AT4G16260.1	10.84/3.93
LOC8055009	Tpd1 protein homolog 1 isoform X1	AT4G32110.1	8.74/8.33
LOC8079922	Photosystem Ii 10 Kda polypeptide, chloroplastic	NA	8.27/3.95
LOC8086360	Glutaredoxin-C1	AT3G21460.1	7.98/5.78
LOC8064774	DNA (cytosine-5)-methyltransferase 1-like	AT4G23160.1	7.49/NA
LOC8056752	Uncharacterized protein Loc8056752	AT2G32020.1	7.17/4.47
LOC110431695	Uncharacterized protein Loc110431695	NA	6.75/NA
LOC8071420	Amino-Acid Permease Bat1 Homolog	AT2G01170.2	6.71/4.02
LOC110431768	5-Pentadecatrienyl resorcinol O-methyltransferase-like	AT4G35160.1	6.69/6.08
LOC8082233	Protein Dmp3	AT4G24310.1	6.55/3.45
LOC8064888	Glycine-rich cell wall structural protein 2	NA	6.47/7.58
LOC8060812	Lecithin-cholesterol acyltransferase-like 1	AT1G27480.1	6.41/2.42
LOC8069518	Glutaredoxin-C10	AT4G15700.1	6.40/10.16
LOC8083139	Alpha-humulene synthase isoform X2	AT5G23960.2	6.19/7.40
LOC110430523	Uncharacterized protein Loc110430523	NA	6.13/2.83
LOC8075318	Uncharacterized protein Loc8075318	NA	6.05/NA
LOC8063193	21 Kda protein-like	NA	6.02/1.85
LOC8067575	Asparagine synthetase [glutamine-hydrolyzing]	AT3G47340.1	5.99/2.96
LOC8079582	Uncharacterized Loc100303807	NA	5.82/3.14
LOC8071734	E3 Ubiquitin-protein ligase Atl23	AT3G10910.1	5.79/5.55
LOC8065780	Basic blue protein	AT5G26330.1	5.78/NA
LOC8076097	Dihydroflavonol 4-reductase	AT5G42800.1	5.69/4.12
LOC110434920	Uncharacterized protein Loc110434920	NA	5.68/3.37

### Characteristics of DEGs

Scanning the RNASeq defined DEGs that are above the statistical cutoff, including those that increased or decreased in both or only in one cultivar or the other, turned up several interesting observations. With a single exception, in all 3,160 cases where the same gene was identified as being differentially expressed in both cultivars, both were either upregulated or both showed downregulation; the exception was a chaperone protein *dnaJ C76*, *chloroplastic isoform X2*, which was upregulated 1.03-fold in QL3 and downregulated 1.19-fold in Theis. As in this case, the vast majority of relative differences for the cultivars, up or down, were within 2-fold. The most extreme cases include a lichenase-2 precursor, which was up 10.8-fold in QL3 and only 3.9-fold in Theis. Lichenases are involved in the hydrolysis of D glucans in plant cell walls during growth, and a lichenase has been shown to inhibit the growth of Fusarium *in vitro* (Mirakhorli et al., 2019). A lichenase has also been shown to be differentially upregulated in potatoes in response to *Alternaria alternata* (Brouwer et al., 2020). An opposite effect was seen for a peroxidase mRNA that was 9.1-fold increase in Theis but only 3.8-fold increase in QL3, implying a greater level of the oxidative burst in the susceptible cultivar, which suggests a greater degree of pathogen ingress may occur.

Searching for genes that based on previous results, were predicted to show induced levels of expression within 24 hpi gave negative or inconsistent results. For example, expression of a chitinase-2 gene was up 2.6-fold in QL3 and 1.9 in Theis but mRNA levels for chitinase-6 were down 1.8 and 2.0-fold, respectively in the two cultivars. QL3 also had a chitinase-1 that was up 2.7 and a chitinase-11 that was down-1.1, neither of which were among the DEGs for Thies. While this could indicate activation of a defense-activated chitinase concomitant with inactivation of a different chitinase, there is not sufficient information on the specific genes to assign those roles. For phenylalanine ammonia lyase, the first step required to make the sorghum phytoalexin apegeninidin, mRNA levels actually decreased 2.8 and 3.5-fold in QL3 and Theis, respectively. Flavone synthase, required to make luteolinidin, another antifungal that originates from a shared precursor (Poloni and Schirawski, 2014) did not appear, but the expression of a downstream gene that encodes isoflavone-3-hydroxylase was downregulated by factors of 3.3 (QL3) and 6.0 (Theis). No evidence for altered expression of pathogenesis response gene *PR10*, which is often found in sorghum soon after inoculation, was detected. However, PR-4 was up three in both as was PR3, while three PR1 genes upregulated between 2 and 3-fold in Theis were not among the DEGs seen in QL-3. Of the genes that showed dramatic decreases in specific gene expression in one or both cultivars, the only genes recognized as having a potential role in host defense were the previously listed flavonol synthase/flavanone 3-hydroxylase and a probable cinnamyl alcohol dehydrogenase 5 that functions in the PAL pathway. Its expression was down 9.3-fold in QLE and 5.2-fold in Theis.

Many of the 3,160 DEGs showing significant changes between the zero time and 24-hpi samples for both cultivars have potential roles in host defense, where even small changes in expression can have enormous downstream consequences. This includes genes with LRR domains that are characteristic of cloned resistance genes, genes that function in signal transduction pathways, and transcription factors, including zinc-finger proteins or WRKY motifs, among others (Table 4). As a consequence, we elected to take advantage of various gene function classification systems and pathway enrichment analysis to seek further understanding.

**Table 4:** Differentially expressed genes with transcription factors upregulated in resistant genotype QL3 and corresponding expression in Theis.

Gene ID	TF name	QL3 (Log_2_FC)	Theis (Log_2_FC)
LOC8057890	GARP-G2-like	5.022471468	3.407295773
LOC110434506	bHLH	4.57456493	3.224728017
LOC8060845	WRKY	4.368436165	3.665955486
LOC110429792	NAC	4.253140922	NA
LOC8081124	MYB-related	4.236286193	2.374842293
LOC8068183	MYB	3.796296652	4.563927241
LOC8064748	WRKY	3.742064459	2.560542823
LOC8072366	NAC	3.509181013	2.760633958
LOC8069635	C2C2-LSD	3.447913101	1.27486572
LOC8064504	LOB	3.436689005	3.096194484
LOC8065521	TCP	3.316294574	3.362707674
LOC8055957	AP2/ERF-ERF	3.144218827	1.738455777
LOC8068938	EIL	3.012716092	4.094994129
LOC8082008	AP2/ERF-ERF	2.977434502	NA
LOC8078627	bHLH	2.941957689	NA
LOC8075863	GRAS	2.870628523	2.177267148
LOC8066792	B3	2.843024947	1.745466608
LOC8057846	MYB-related	2.840415992	NA
LOC8061040	AP2/ERF-ERF	2.771432866	NA
LOC8084855	WRKY	2.703193683	3.173130372
LOC110434014	AP2/ERF-ERF	2.683231661	4.455836791
LOC8058462	NAC	2.55387135	NA
LOC8085025	NAC	2.504405907	2.364131657

### Pathway Enrichment Analysis

**Figure 2:**
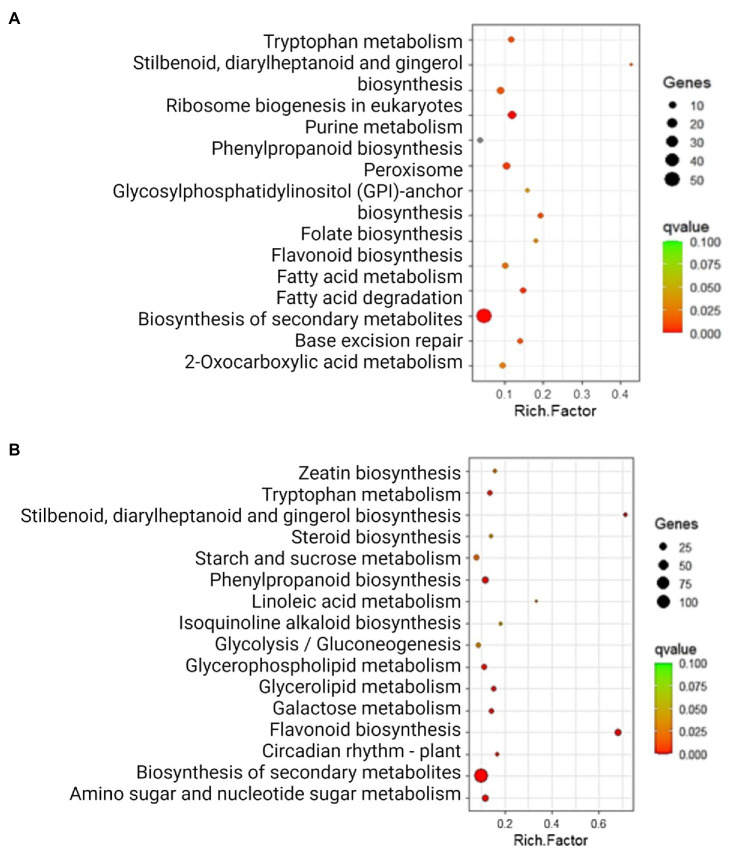
Significantly enriched (*q* < 0.05) KEGG pathways among the DEGs exclusively upregulated **(A)** and downregulated **(B)** in resistant genotype QL3. Rich factor is the ratio of the number of DEGs to the total gene number in a pathway. Here, the *q*-value is a corrected *p*-value. The color and size of the dots represent the range of *q*-value, and the number of DEGs mapped to the indicated pathways, respectively.

**Figure 3:**
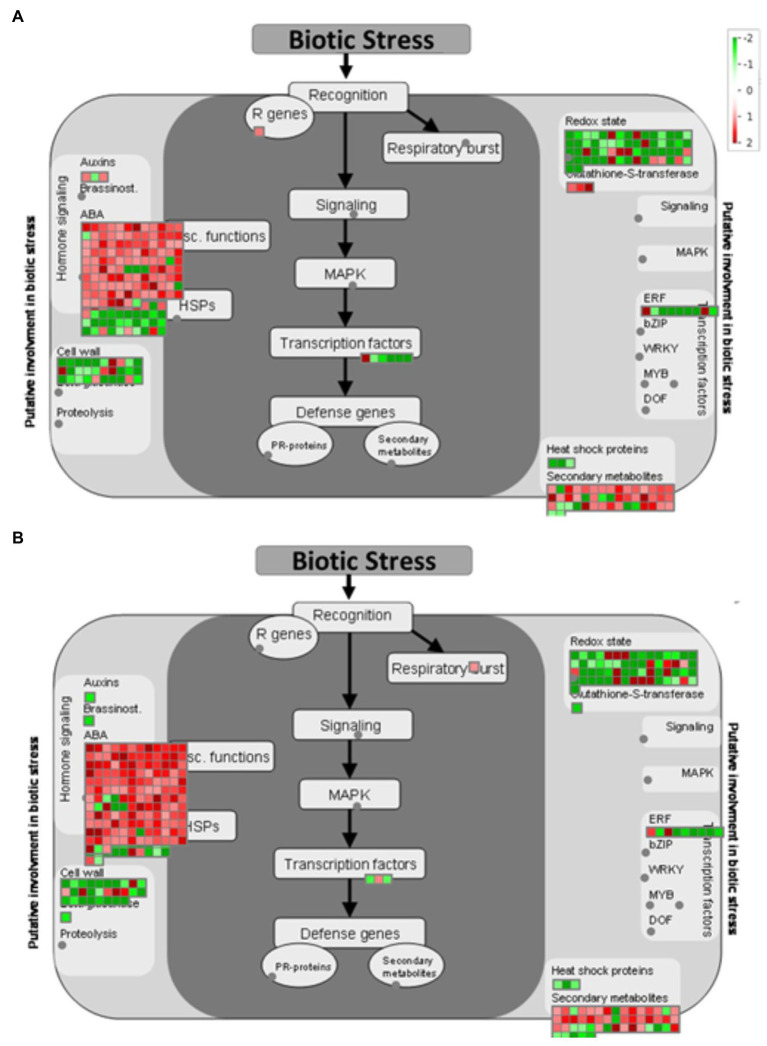
MapMan visualization shows significant (FDR adjusted *p* < 0.05 and log_2_Fold > 1) DEGs mapped to the biotic stress pathway from resistant genotype QL3 **(A)** and susceptible genotype Theis **(B)** upon Johnsongrass anthracnose infection. DEGs are binned to MapMan functional categories and values are represented as log 2-fold change values. Red represents upregulated DEGs and green represents downregulated DEGs.

**Figure 4:**
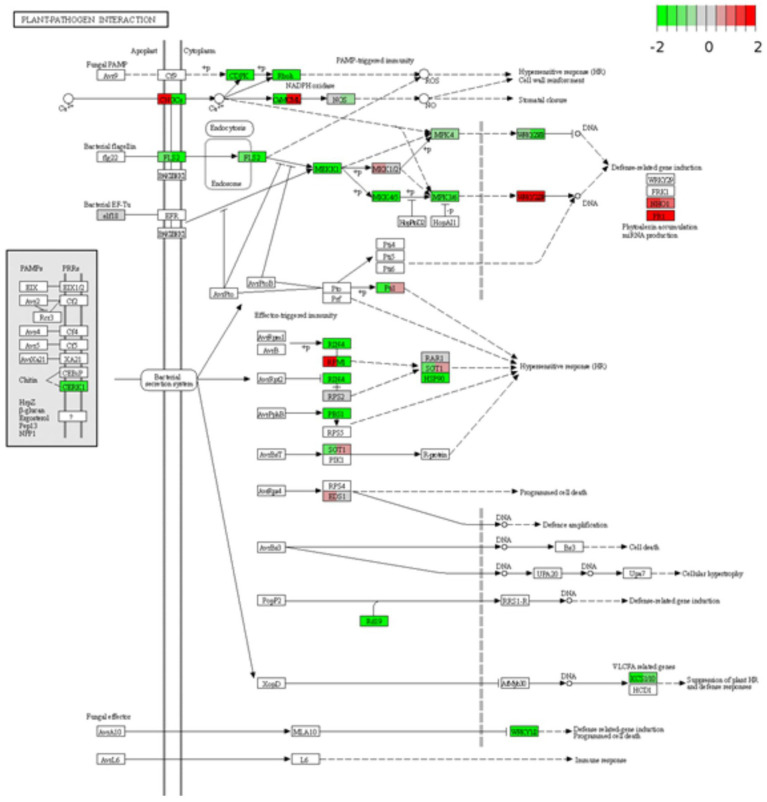
Differentially expressed genes are involved in plant-pathogen interactions. Expression values of the DEGs are presented as log_2_Fold Change > 1 (red for upregulated, green for downregulated). The vertical columns represent the gene expression for samples QL3 24 h post-inoculation (hpi) and Theis 24 hpi from left to right.

After identifying DEGs *via* sequence alignment to the annotated sorghum genome, pathway enrichment analysis was used to compare the relative numbers of genes from multiple biological pathways among the genes identified as having differential expression in QL3 compared to the Theis (Figure 2). MapMan analysis revealed several genes for biotic stress differentially expressed in the two hosts 24 hpi, including genes related to cell wall metabolism, redox function, and hormone responses (Figure 3). Mapping the DEGs to the pathways of plant-pathogen interactions reveled important genes differentially expressed in QL3 vs. Theis in 24 hpi (Figure 4).

### DEGs for Disease Resistance

**Figure 5:**
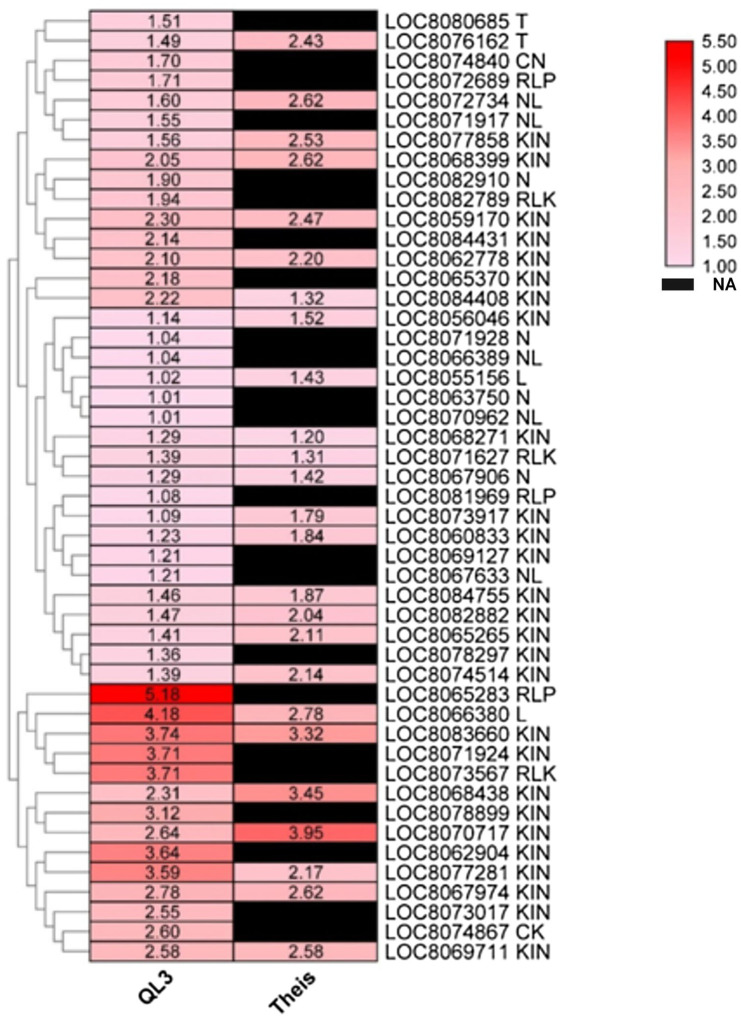
Heatmap shows the expression of DEGs for plant disease resistance in resistant (QL3) and susceptible (Theis) genotypes upon Johnsongrass anthracnose infection. Plant disease resistance genes (R-genes) and their classes are included in the gene name. Gene expression values are presented as log_2_Fold Change. “NA” denotes no DEGs found in a respective sample.

Plant resistance genes (R-genes) encoded proteins initiate pathways leading to biotic disease resistance in plants (Ma et al., 2009). There were 48 R-genes upregulated in QL3 in response to infection (Figure 5). This includes CK (1), CN (1), L (2), T (2), RLK (3), RLP (3), N (4), NL (5), KIN (27), and their expression levels ranged from 1 to 5-fold in QL3.

### GO Enrichment Analysis of DEGs

**Figure 6:**
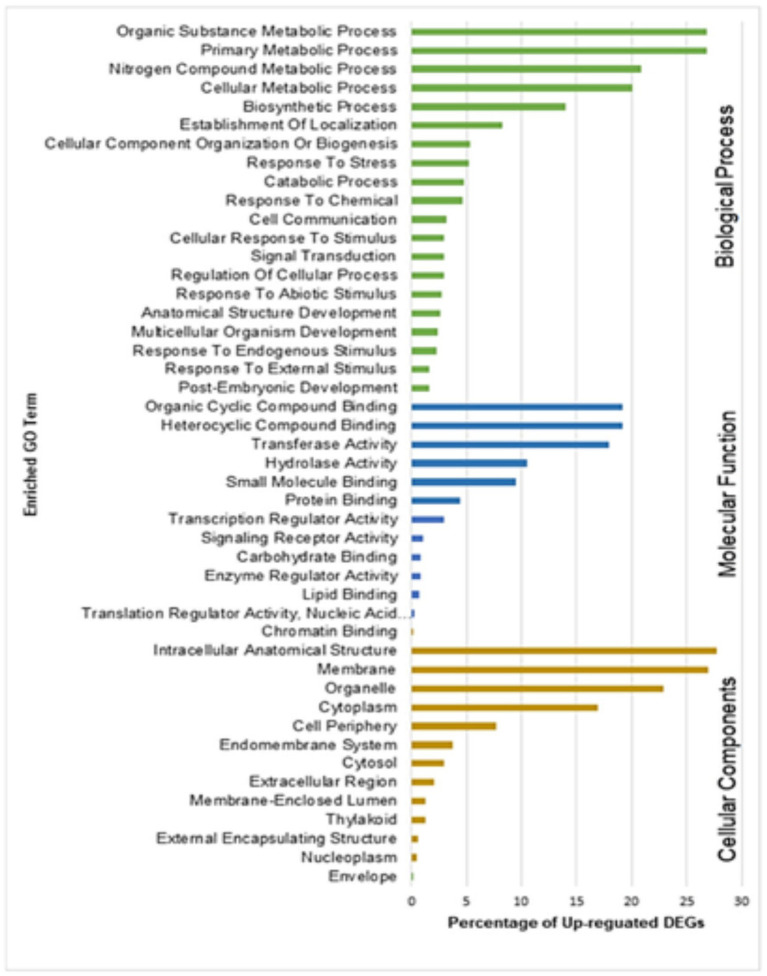
Gene ontology (GO) enrichment analysis of DEGs upregulated in resistant genotype QL3 vs. Theis.

Gene Ontology (GO) enrichment analysis helps to understand DEGs regulation under the three important categories: biological processes, molecular functions, and cellular components. GO enrichment analysis of upregulated DEGs from resistant genotype (QL3) revealed the functional categories activated with the Johnsongrass anthracnose infection (Figure 6). The significantly enriched biological processes upregulated in QL3 upon infection included several stress responses processes and plant development processes. This includes response to stress (103 DEGs), response to abiotic stimulus (54 DEGs), cellular response to stimulus (58 DEGs), signal transduction (58 DEGs), cell communications (64 DEGs), anatomical structural development (53 DEGs), and post-embryonic development (31 DEGs).

## Discussion

As indicated previously, past work has shown that initial responses to exposure to *Colletotrichum* induce defense response reactions in both resistant and susceptible hosts. Examples include visual production of PAL-derived phytoalexins (Lo et al., 1999) and increased levels of mRNAs for phytoalexin pathway enzymes such as PAL and chalcone synthase (Cui et al., 1996). In addition, cytological studies showed that spore penetration occurs within 24 h in both the resistant and susceptible hosts, but that spread from the initially penetrated cells was not seen in the resistant host (Wharton and Julian, 1996). Thus, it has been assumed that resistant cultivars must either trigger an earlier or higher level of activation of genes critical for the defense response or that resistance involves genes other than those for which similar levels of induction occur in both the resistant and susceptible hosts. Consequently, it was a surprise to see that several of the genes such as PAL that were expected to be induced in both cultivars actually decreased in expression. A potential explanation besides the fact that different cultivars with different forms of resistance were used is that the earlier studies were based on northern blots or qPCR primers that were not specific to gene family members, some of which may respond to different signals. Since fold level changes do not reflect actual mRNA levels, only changes from the control, plus, and minus DEG values may not reflect actual levels of expression. There may also be cases when short sequences from conserved regions of gene family members get assigned to different genes in mapping to the genome.

Even so, RNAseq data provide an excellent opportunity to explore the potential molecular basis for resistance further. For example, as shown in Table 4, 18 of 23 genes identified as transcription factors were more highly expressed in QL3 24 hpi than in Thies. Also, mRNAs for a DNA cytosine methylase, an enzyme that leads to cytosine methylation in the di- or trinucleotide sequences CG or CXG, were highly enriched only in QL3. The epigenetic effect of ^5^C methylation in promoters is associated with turning off transcription and is also a downstream consequence of PTGS, a mechanism of combating RNA viruses in plants (Deleris et al., 2016). Several of the significantly upregulated genes detected are protein kinases that typically activate proteins that function in signal transduction pathways that function between recognizing pathogen elicitors (Antolín-Llovera et al., 2012) and are reflected in Figure 3 as map kinases. Basic blue proteins in plants are copper-containing plantacyanins that are regulated *via* conserved microRNA408 (miR408), which is present in sorghum (Zhang et al., 2011). Although, miR408 was not identified among the upregulated miRNAs detected by RNAseq in anthracnose-resistant sorghum cultivar SC383 following inoculation with *C. sublineolum* (Fu et al., 2020), a miR408 induced basic blue protein in wheat has been demonstrated to have a role in resistance to stripe rust (*Puccinia striiformis* f. sp. *tritici*; Feng et al., 2013). The ent-cassadiene C2-hydroxylases function in pathways for converting geranylgeranyl-PP into a variety of compounds, including terpene-based phytoalexins. In rice, the expression of these enzymes is regulated by a microRNA encoded by *DCL1*; inactivation of *DCL1* altered the production of terpene-based phytoalexins in rice, thus increasing sensitivity to rice blast caused by *Magnaporthe oryzae* and another fungal pathogen *Fusarium fujikuroi* (Salvador-Guirao et al., 2019). The presence of these genes in the upregulated transcripts implies that these genes may also play a role in multigenic pathogen resistance in QL3.

The list of DEGs that show similar up or downregulation in both hosts (Supplementary Table 3) may also reveal new avenues important to active host defense. As an example, lichenase 2 precursor was at the top of the list of upregulated genes in both hosts. While lichenase [an endo-(1−>4)-beta-glucanase] has not been reported as a defense-related gene in sorghum, it has also been found to be highly induced in banana 27 hpi with *Fusarium oxysporum* f. sp. cubense in a race-specific manner (Li et al., 2013). Chitinase 2 was upregulated in both hosts, but chitinase six expressions were reduced by nearly the same fold level, implicating chitinase 2 as more likely to be active host defense. Furthermore, there are a number of LRR and F box proteins, some of which are upregulated and others downregulated that may be important: leucine-rich repeat (LRR) regions of a protein allow interactions between proteins, including those of host and pathogen origin. Most known resistance genes in plants have an LRR domain (Gururani et al., 2012). F-box proteins are best known for functioning in growth regulation and development guided by hormone signals, but at least one is known to be involved in cell death triggered by pathogen recognition as part of the (HR; van den Burg et al., 2008).

We found 3,150 DEGs downregulated in susceptible cultivar (Theis), and these genes are important to study the functions affected by Johnsongrass anthracnose infection in Theis. Complete list of up/downregulated DEGs from Theis available in Supplementary Table 2. Interestingly, early nodulin-93 was the most downregulated gene in the susceptible cultivar upon Johnsongrass anthracnose infection. This gene was downregulated more than 10-fold in Theis. This gene is essential for maintaining cell structure and nitrogen-use efficiency in plants (Okubara et al., 2000; Bi et al., 2009). The downregulation of this gene might have affected the growth and health of susceptible cultivar.

### Enrichment Analysis

Results gained from the various bioinformatics resources based on the proteins encoded by the enriched mRNAs are also informative. The databases used classify functions in cellular processes (GO), pathway components (KEGG and BioCys), and evolutionary relationships (Panther). As seen in Figure 2, the largest category of upregulated genes in QL3 involves secondary metabolites, which would include the flavonoid and PAL pathway genes for making sorghums’ antimicrobial compounds and coumarins that are listed. A surprise in the list includes camalexin, a defense compound made by *Arabidopsis thaliana*, but it turns out that the pathway components are analogous to the enzymes sorghum uses to make a similar defense compound, dhurrin (Klein et al., 2013). It is also of interest that the highest relative expression in QL3 (Supplementary Table 1) was an enzyme involved in benzoate biosynthesis. While benzoate is widely used as a preservative, some sorghum fungal have a Cyt P450 enzyme that can detoxify them (Korošec et al., 2014). However, sorghum also makes cinnamic acid derivatives that can inhibit the detoxification (Nguyen et al., 2015).

### Notable DEGs Based on the MapMan Biotic Stress Pathway

A sorghum equivalent of an *Arabidopsis* gamma-Tubulin ring complex (gamma-TuRC) gene was found to be 1.2-fold upregulated in resistant cultivar QL3 in response to the infection. This protein is important for the plant cytoskeleton, including microtubule organization and elongation (Zeng et al., 2009). Plant cytoskeleton provides a major defense against invading fungal pathogens and is the major regulator and target for biotic interaction in plants (Takemoto and Hardham, 2004). Additionally, another protein, MT plus-end-tracking protein (EB1), is expressed at 2.3-fold higher 24 hpi than immediately after inoculation in QL3. This protein is also important for the microtubule bundling and organization in plants, which is essential for the plants biotic stress response and plant growth (Molines et al., 2018). The results infer that microtubule organization played an important role in QL3 in biotic stress response.

Calcium cation channel (DMI1/Pollux Castor) expressed 1.3-fold only in QL3 with anthracnose infection. DMI1 has been shown to interact with Ca^2+^−permeable cyclic nucleotide-gated (CNGC) channels at the nuclear envelopes. DM1proteins function as ligand-gated K^+^ channels that can modulate the membrane potential across the nuclear envelope and work together with CNGC channels to modulate nuclear Ca^2+^ release. CNGCs play a crucial role in pathways related to cellular ion homeostasis, development, and as a “guard” in defense against biotic stress (Jha et al., 2016). Sorghum cDNAs that mapped to *Arabidopsis* CNGCs were expressed about 3.8-fold higher in QL3 in response to infection. CNGCs play an important role in the innate immune response in plants (Ma et al., 2009). Three CNGCs (CNGC1, CNGC 4, and CNGC 9) were found to be upregulated (3.8, 2.3, and 1.3-fold, respectively) in QL3 in response to infection when compared to Theis (Supplementary Table 1).

RPM1 is a plant immune receptor that explicitly recognizes pathogen-released effectors to activate effector-triggered immunity (ETI) in *Arabidopsis thaliana* (Grant et al., 1995). Sorghum genes equivalent to *Arabidopsis* RPM1 were more highly expressed in resistant cultivar QL3 than the susceptible Theis (Figure 3). Three different sorghum homologs of *Arabidopsis* RPM1 were observed to be uniquely upregulated in the QL3 file (1.6, 1.6, and 1.3-fold, respectively), and two others for different *Arabidopsis* RPM genes were up 1.7 and 1.0. Expression of an RPM homolog found in Theis decreased 6.1-fold. Overall, these observations clarify the value of grouping genes by GO and functional categories based on RNASeq data to reveal genes that may play a role in hast diseases that would be difficult to detect on a one gene at a time basis.

## Conclusion

We have analyzed the transcriptome changes associated with the inoculation of *C. sublineola* in two inbred sorghum cultivars (QL3 and Theis) known to differ in their anthracnose disease development. Differential gene expression analysis identified over 3,000 specific genes in both cultivars as showing significant changes in expression following inoculation. Gene ontology and pathway enrichment analysis identified upregulation of 23 transcription factor encoding genes as well as genes involved in the production of secondary metabolites, which are part of a typical host defense reaction.

## Data Availability Statement

The raw RNA sequencing reads generated from the current study have been deposited at NCBI under the BioProject accession number PRJNA736032.

## Author Contributions

CM and UR conceived the study and designed the research experiments. PN, EA, UR, RP, CM, and LP conducted the experiments, analyzed the data, and wrote the manuscript. All authors contributed to the article and approved the submitted version.

## Conflict of Interest

The authors declare that the research was conducted in the absence of any commercial or financial relationships that could be construed as a potential conflict of interest.

## Publisher’s Note

All claims expressed in this article are solely those of the authors and do not necessarily represent those of their affiliated organizations, or those of the publisher, the editors and the reviewers. Any product that may be evaluated in this article, or claim that may be made by its manufacturer, is not guaranteed or endorsed by the publisher.

## Footnotes

^1^ http://www.morrislab.org/data

^2^ https://www.biobam.com/omicsbox/

## References

[ref1] Ahn E. Hu Z. Perumal R. Prom L. K. Odvody G. Upadhyaya H. D.. (2019). Genome wide association analysis of sorghum mini core lines regarding anthracnose, downy mildew, and head smut. PLoS One 14:e0216671. 10.1371/journal.pone.0216671, PMID: 31086384 PMC6516728

[ref2] Ahn E. Odvody G. Prom L. K. Magill C. (2020). Late growth stages of johnsongrass can act as an alternate host of *Colletotrichum sublineola*. Plant Health Prog. 21, 60–62. 10.1094/PHP-11-19-0084-RS

[ref3] Anders S. Pyl P. T. Huber W. (2015). HTSeq—a Python framework to work with high-throughput sequencing data. Bioinformatics 31, 166–169. 10.1093/bioinformatics/btu638, PMID: 25260700 PMC4287950

[ref4] Antolín-Llovera M. Ried M. K. Binder A. Parniske M. (2012). Receptor kinase signaling pathways in plant-microbe interactions. Annu. Rev. Phytopathol. 50, 451–473. 10.1146/annurev-phyto-081211-173002, PMID: 22920561

[ref5] Bi Y. M. Kant S. Clark J. Gidda S. Ming F. Xu J.. (2009). Increased nitrogen-use efficiency in transgenic rice plants over-expressing a nitrogen-responsive early nodulin gene identified from rice expression profiling. Plant Cell Environ. 32, 1749–1760. 10.1111/j.1365-3040.2009.02032.x, PMID: 19682292

[ref6] Bolger A. Giorgi F. (2014). Trimmomatic: a flexible read trimming tool for illumina NGS data. Bioinformatics 30, 2114–2120. 10.1093/bioinformatics/btu170, PMID: 24695404 PMC4103590

[ref201] Broadhead D. M. Freeman K. C. Coleman O. H. Zummo N. (1978). Registration of Theis sweet sorghum (Reg. No. 117). Crop Sci. 18:165.

[ref7] Brouwer S. M. Odilbekov F. Burra D. D. Lenman M. Hedley P. E. Grenville-Briggs L.. (2020). Intact salicylic acid signalling is required for potato defence against the necrotrophic fungus Alternaria solani. Plant Mol. Biol. 104, 1–19. 10.1007/s11103-020-01019-6, PMID: 32562056 PMC7417411

[ref8] Casa A. M. Pressoir G. Brown P. J. Mitchell S. E. Rooney W. L. Tuinstra M. R.. (2008). Community resources and strategies for association mapping in sorghum. Crop Sci. 48, 30–40. 10.2135/cropsci2007.02.0080

[ref9] Cooper E. A. Brenton Z. W. Flinn B. S. Jenkins J. Shu S. Flowers D.. (2019). A new reference genome for *Sorghum bicolor* reveals high levels of sequence similarity between sweet and grain genotypes: implications for the genetics of sugar metabolism. BMC Genomics 20:420. 10.1186/s12864-019-5734-x, PMID: 31133004 PMC6537160

[ref10] Cuevas H. E. Prom L. K. Cooper E. A. Knoll J. E. Ni X. (2018). Genome-wide association mapping of anthracnose (*Colletotrichum sublineolum*) resistance in the US Sorghum association panel. Plant Genome 11:170099. 10.3835/plantgenome2017.11.0099, PMID: 30025025 PMC12962439

[ref11] Cuevas H. E. Prom L. K. Cruet-Burgos C. M. (2019). Genome-wide association mapping of anthracnose (*Colletotrichum sublineolum*) resistance in NPGS Ethiopian sorghum germplasm. G3 9, 2879–2885. 10.1093/bioinformatics/bts635, PMID: 31289022 PMC6723129

[ref12] Cui Y. Magill J. Frederiksen R. Magill C. (1996). Chalcone synthase and phenylalanine ammonia-lyase mRNA levels following exposure of sorghum seedlings to three fungal pathogens. Physiol. Mol. Plant Pathol. 49, 187–199. 10.1006/pmpp.1996.0048

[ref13] Deleris A. Halter T. Navarro L. (2016). DNA methylation and demethylation in plant immunity. Annu. Rev. Phytopathol. 54, 579–603. 10.1146/annurev-phyto-080615-100308, PMID: 27491436

[ref14] Dobin A. Davis C. A. Schlesinger F. Drenkow J. Zaleski C. Jha S.. (2013). STAR: ultrafast universal RNA-seq aligner. Bioinformatics 29, 15–21. 10.1093/bioinformatics/bts635, PMID: 23104886 PMC3530905

[ref15] Feng H. Zhang Q. Wang Q. Wang X. Liu J. Li M.. (2013). Target of tae-miR408, a chemocyanin-like protein gene (TaCLP1), plays positive roles in wheat response to high-salinity, heavy cupric stress and stripe rust. Plant Mol. Biol. 83, 433–443. 10.1007/s11103-013-0101-9, PMID: 23864359

[ref16] Fletcher R. A. Varnon K. M. Barney J. N. (2020). Climate drives differences in the germination niche of a globally distributed invasive grass. J. Plant Ecol. 13, 195–203. 10.1093/jpe/rtz062

[ref17] Fu F. Girma G. Mengiste T. (2020). Global mRNA and microRNA expression dynamics in response to anthracnose infection in sorghum. BMC Genomics 21, 1–16. 10.1186/s12864-020-07138-0, PMID: 33143636 PMC7641857

[ref18] Grant M. R. Godiard L. Straube E. Ashfield T. Lewald J. Sattler A.. (1995). Structure of the *Arabidopsis* RPM1 gene enabling dual specificity disease resistance. Science 269, 843–846. 10.1126/science.7638602, PMID: 7638602

[ref19] Gururani M. A. Venkatesh J. Upadhyaya C. P. Nookaraju A. Pandey S. K. Park S. W. (2012). Plant disease resistance genes: current status and future directions. Physiol. Mol. Plant Pathol. 78, 51–65. 10.1016/j.pmpp.2012.01.002

[ref20] Jha S. K. Sharma M. Pandey G. K. (2016). Role of cyclic nucleotide gated channels in stress management in plants. Curr. Genomics 17, 315–329. 10.2174/1389202917666160331202125, PMID: 27499681 PMC4955031

[ref21] Klein A. P. Anarat-Cappillino G. Sattely E. S. (2013). Minimum set of cytochromes P450 for reconstituting the biosynthesis of camalexin, a major *Arabidopsis* antibiotic. Angew. Chem. 125, 13870–13873. 10.1002/anie.201307454, PMID: 24151049 PMC3867539

[ref22] Korošec B. Sova M. Turk S. Kraševec N. Novak M. Lah L.. (2014). Antifungal activity of cinnamic acid derivatives involves inhibition of benzoate 4-hydroxylase (CYP 53). J. Appl. Microbiol. 116, 955–966. 10.1111/jam.12417, PMID: 24314266

[ref23] Li C. Shao J. Wang Y. Li W. Guo D. Yan B.. (2013). Analysis of banana transcriptome and global gene expression profiles in banana roots in response to infection by race 1 and tropical race 4 of *Fusarium oxysporum* f. sp. cubense. BMC Genomics 14:851. 10.1186/1471-2164-14-851, PMID: 24304681 PMC4046742

[ref24] Lo S.-C. C. De Verdier K. Nicholson R. L. (1999). Accumulation of 3-deoxyanthocyanidin phytoalexins and resistance to *Colletotrichum sublineolum* in sorghum. Physiol. Mol. Plant Pathol. 55, 263–273. 10.1006/pmpp.1999.0231

[ref25] Love M. Anders S. Huber W. (2014). Differential analysis of count data–the DESeq2 package. Genome Biol. 15:550. 10.1186/s13059-014-0550-8 25516281 PMC4302049

[ref26] Luo W. Pant G. Bhavnasi Y. K. Blanchard S. G. Jr. Brouwer C. (2017). Pathview web: user friendly pathway visualization and data integration. Nucleic Acids Res. 45, W501–W508. 10.1093/nar/gkx372, PMID: 28482075 PMC5570256

[ref27] Ma W. Smigel A. Verma R. Berkowitz G. A. (2009). Cyclic nucleotide gated channels and related signaling components in plant innate immunity. Plant Signal. Behav. 4, 277–282. 10.4161/psb.4.4.8103, PMID: 19794842 PMC2664486

[ref28] Mirakhorli N. Norolah Z. Foruzandeh S. Shafizade F. Nikookhah F. Saffar B.. (2019). Multi-function plant defensin, antimicrobialand heavy metal adsorbent peptide. Iran. J. Biotechnol. 17, 43–49. 10.29252/ijb.1562 PMC7080970 32195280

[ref29] Molines A. Marion J. Chabout S. Besse L. Dompierre J. Mouille G.. (2018). EB1 contributes to microtubule bundling and organization, along with root growth, in *Arabidopsis thaliana*. Biol. Open 7:bio030510. 10.1242/bio.030510, PMID: 29945874 PMC6124560

[ref30] Morris G. P. Ramu P. Deshpande S. P. Hash C. T. Shah T. Upadhyaya H. D.. (2013). Population genomic and genome-wide association studies of agroclimatic traits in sorghum. Proc. Natl. Acad. Sci. 110, 453–458. 10.1073/pnas.1215985110, PMID: 23267105 PMC3545811

[ref31] Nguyen P.-H. Zhao B. T. Lee J. H. Kim Y. H. Min B. S. Woo M. H. (2015). Isolation of benzoic and cinnamic acid derivatives from the grains of sorghum bicolor and their inhibition of lipopolysaccharide-induced nitric oxide production in RAW 264.7 cells. Food Chem. 168, 512–519. 10.1016/j.foodchem.2014.06.119, PMID: 25172742

[ref32] Ohadi S. Hodnett G. Rooney W. Bagavathiannan M. (2017). Gene flow and its consequences in *Sorghum* spp. Crit. Rev. Plant Sci. 36, 367–385. 10.1080/07352689.2018.1446813

[ref33] Okubara P. A. Fujishige N. A. Hirsch A. M. Berry A. M. (2000). Dg93, a nodule-abundant mRNA of *Datisca glomerata* with homology to a soybean early nodulin gene. Plant Physiol. 122, 1073–1080. 10.1104/pp.122.4.1073, PMID: 10759502 PMC58941

[ref34] Osuna-Cruz C. M. Paytuvi-Gallart A. Di Donato A. Sundesha V. Andolfo G. Aiese Cigliano R.. (2018). PRGdb 3.0: a comprehensive platform for prediction and analysis of plant disease resistance genes. Nucleic Acids Res. 46, D1197–D1201. 10.1093/nar/gkx1119, PMID: 29156057 PMC5753367

[ref35] Paterson A. H. Kong W. Johnston R. M. Nabukalu P. Wu G. Poehlman W. L.. (2020). The evolution of an invasive plant, *Sorghum halepense* L.(‘*Johnsongrass*’). Front. Genet. 11:317. 10.3389/fgene.2020.00317, PMID: 32477397 PMC7240026

[ref36] Poloni A. Schirawski J. (2014). Red card for pathogens: phytoalexins in sorghum and maize. Molecules 19, 9114–9133. 10.3390/molecules19079114, PMID: 24983861 PMC6271655

[ref37] Prom L. K. Ahn E. Isakeit T. Magill C. (2019). GWAS analysis of sorghum association panel lines identifies SNPs associated with disease response to Texas isolates of Colletotrichum sublineola. Theor. Appl. Genet. 132, 1389–1396. 10.1007/s00122-019-03285-5, PMID: 30688991

[ref38] Prom L. Perumal R. Erattaimuthu S. Little C. No E. Erpelding J.. (2012). Genetic diversity and pathotype determination of Colletotrichum sublineolum isolates causing anthracnose in sorghum. Eur. J. Plant Pathol. 133, 671–685. 10.1007/s10658-012-9946-z

[ref39] Prom L. K. Perumal R. Isakeit T. Radwan G. Rooney W. L. Magill C. (2015). The impact of weather conditions on response of Sorghum genotypes to anthracnose (Colletotrichum sublineola) infection. J. Exp. Agric. Int. 6, 242–250. 10.9734/AJEA/2015/14589

[ref41] Salvador-Guirao R. Baldrich P. Tomiyama S. Hsing Y.-I. Okada K. San Segundo B. (2019). OsDCL1a activation impairs phytoalexin biosynthesis and compromises disease resistance in rice. Ann. Bot. 123, 79–93. 10.1093/aob/mcy141, PMID: 30032201 PMC6344094

[ref42] Schwacke R. Ponce-Soto G. Y. Krause K. Bolger A. M. Arsova B. Hallab A.. (2019). MapMan4: a refined protein classification and annotation framework applicable to multi-omics data analysis. Mol. Plant 12, 879–892. 10.1016/j.molp.2019.01.003, PMID: 30639314

[ref43] Storey J. D. Tibshirani R. (2003). Statistical significance for genomewide studies. Proc. Natl. Acad. Sci. 100, 9440–9445. 10.1073/pnas.1530509100, PMID: 12883005 PMC170937

[ref44] Takemoto D. Hardham A. R. (2004). The cytoskeleton as a regulator and target of biotic interactions in plants. Plant Physiol. 136, 3864–3876. 10.1104/pp.104.052159, PMID: 15591444 PMC535820

[ref45] Thakur R. Mathur K. (2000). “Anthracnose,” in Compendium of Sorghum Diseases. eds. Fredericksen R. A. Odvody G. N. (St. Paul: The American Phytopathological Society), 10–12.

[ref46] Thimm O. Bläsing O. Gibon Y. Nagel A. Meyer S. Krüger P.. (2004). MAPMAN: a user-driven tool to display genomics data sets onto diagrams of metabolic pathways and other biological processes. Plant J. 37, 914–939. 10.1111/j.1365-313x.2004.02016.x, PMID: 14996223

[ref47] Upadhyaya H. Pundir R. Dwivedi S. Gowda C. Reddy V. G. Singh S. (2009). Developing a mini core collection of sorghum for diversified utilization of germplasm. Crop Sci. 49, 1769–1780. 10.2135/cropsci2009.01.0014

[ref48] van den Burg H. A. Tsitsigiannis D. I. Rowland O. Lo J. Rallapalli G. Maclean D.. (2008). The F-box protein ACRE189/ACIF1 regulates cell death and defense responses activated during pathogen recognition in tobacco and tomato. Plant Cell 20, 697–719. 10.1105/tpc.107.056978, PMID: 18375657 PMC2329923

[ref49] Vermerris W. Cuevas H. Prom L. Knoll J. (2018). Genomic Dissection of Anthracnose Resistance Response in Sorghum [*Sorghum bicolor* (L.) *Moench*]. Gainesville, FL, United States: Univ. of Florida.

[ref50] Wharton P. Julian A. (1996). A cytological study of compatible and incompatible interactions between Sorghum bicolor and *Colletotrichum sublineolum*. New Phytol. 134, 25–34. 10.1111/j.1469-8137.1996.tb01143.x

[ref51] Xavier K. Mizubuti E. Queiroz M. Chopra S. Vaillancourt L. (2018). Genotypic and pathogenic diversity of *Colletotrichum sublineola* isolates from Sorghum (*Sorghum bicolor*) and Johnsongrass (*S. halepense*) in the Southeastern United States. Plant Dis. 102, 2341–2351. 10.1094/PDIS-04-18-0562-RE, PMID: 30199327

[ref52] Zeng C. T. Lee Y.-R. J. Liu B. (2009). The WD40 repeat protein NEDD1 functions in microtubule organization during cell division in *Arabidopsis thaliana*. Plant Cell 21, 1129–1140. 10.1105/tpc.109.065953, PMID: 19383896 PMC2685624

[ref53] Zhang L. Zheng Y. Jagadeeswaran G. Li Y. Gowdu K. Sunkar R. (2011). Identification and temporal expression analysis of conserved and novel microRNAs in Sorghum. Genomics 98, 460–468. 10.1016/j.ygeno.2011.08.005, PMID: 21907786

