# Supplementary figures and images for: RNA-Sequencing in Resistant (QL3) and Susceptible (Theis) Sorghum Cultivars Inoculated With Johnsongrass Isolates of Colletotrichum sublineola
Source: Front Genet. 2021 Aug 11;12:722519. doi: 10.3389/fgene.2021.722519 (PMC8385561; doi:10.3389/fgene.2021.722519)

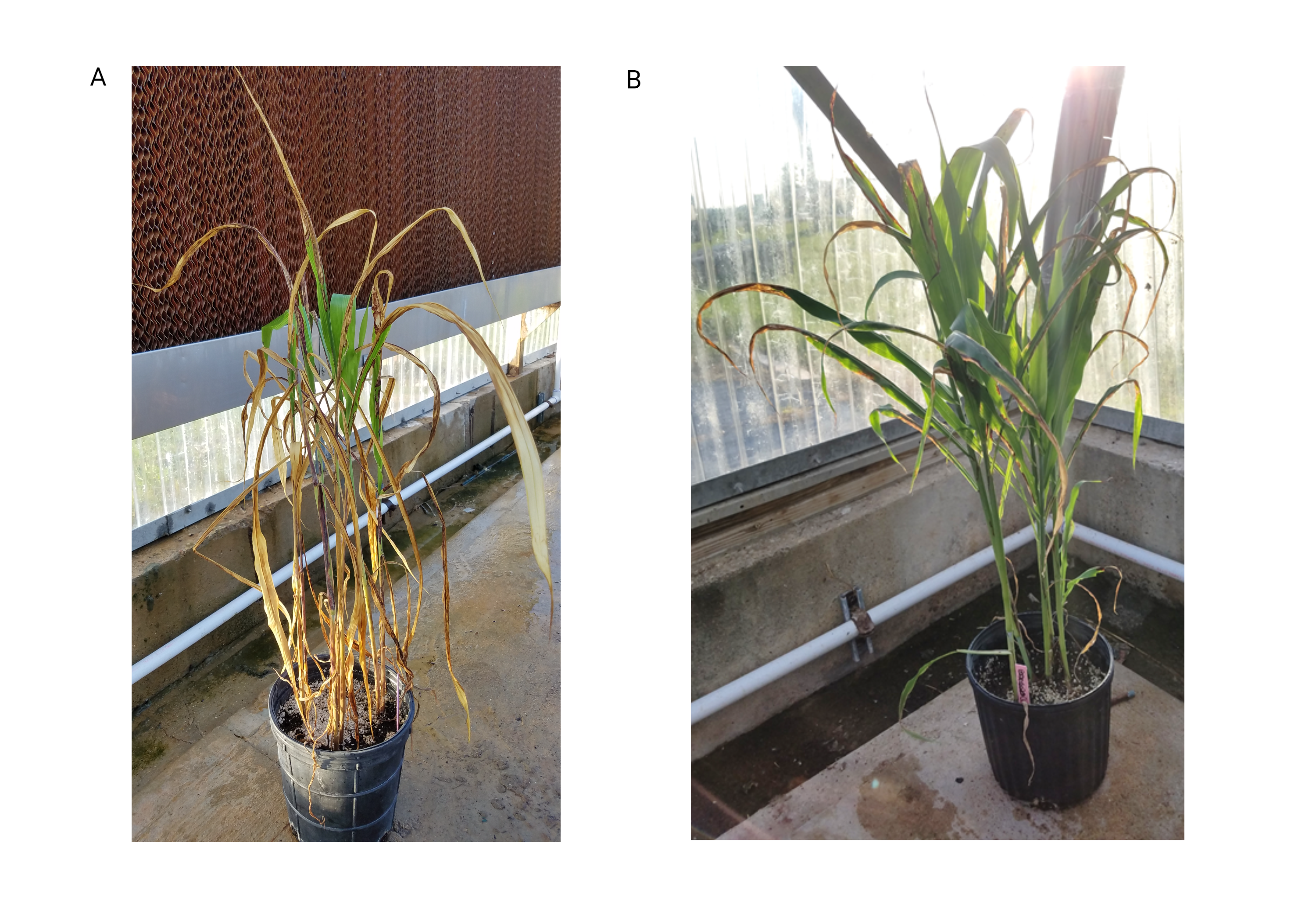

Supplement: Supplementary file 1 [file Image_1.PNG]
